# Supplementary figures and images for: A statistical framework to identify cell types whose genetically regulated proportions are associated with complex diseases
Source: PLoS Genet. 2023 Jul 31;19(7):e1010825. doi: 10.1371/journal.pgen.1010825 (PMC10414598; doi:10.1371/journal.pgen.1010825)

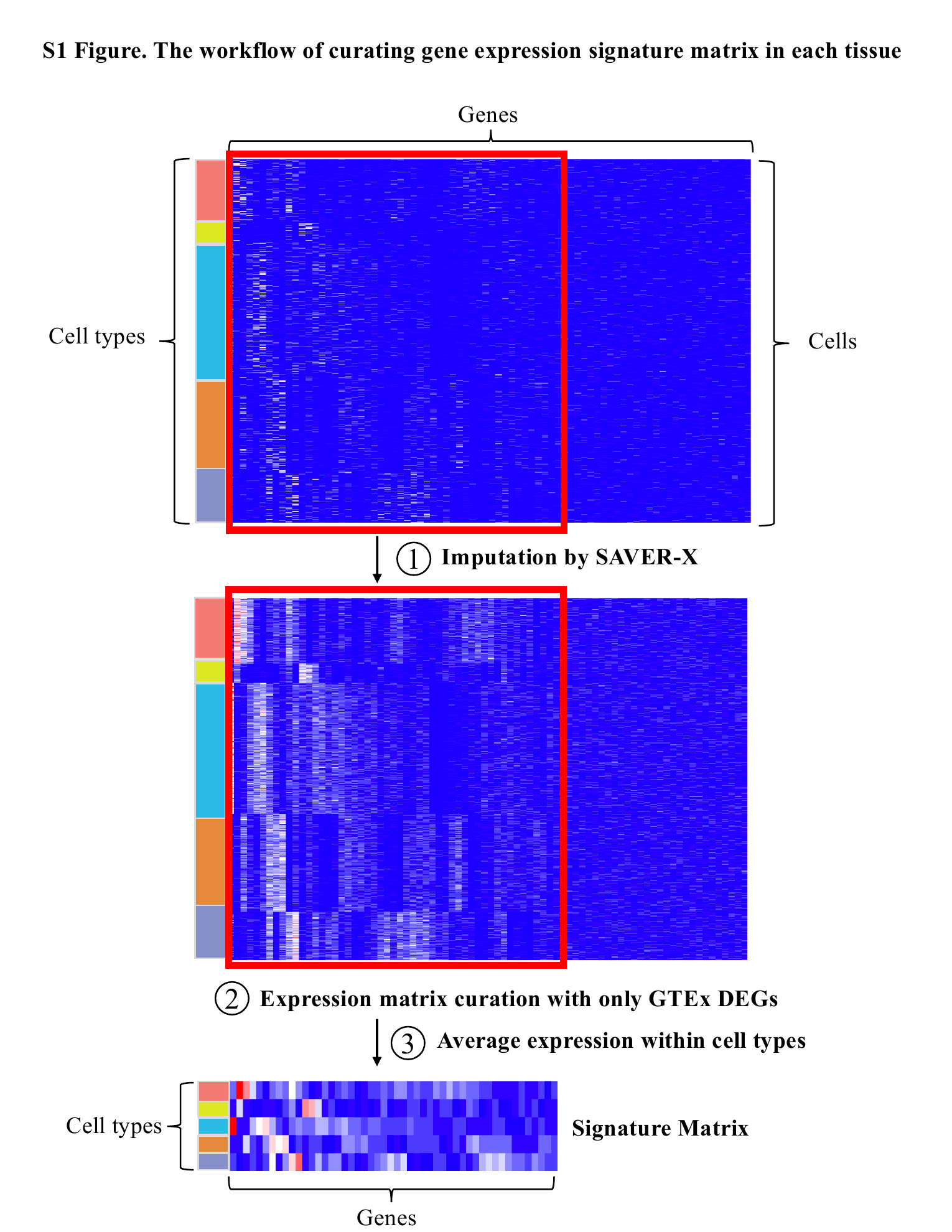

Supplement: S1 Fig — Single cell data across multiple cell types in a given tissue are firstly imputed by SAVER-X and then significant differentially expressed (DE) genes are identified based on cell-type level DE analysis. Finally, for those identified DE genes, their average gene expression levels are computed within each cell type. (TIFF) [file pgen.1010825.s001.tiff]

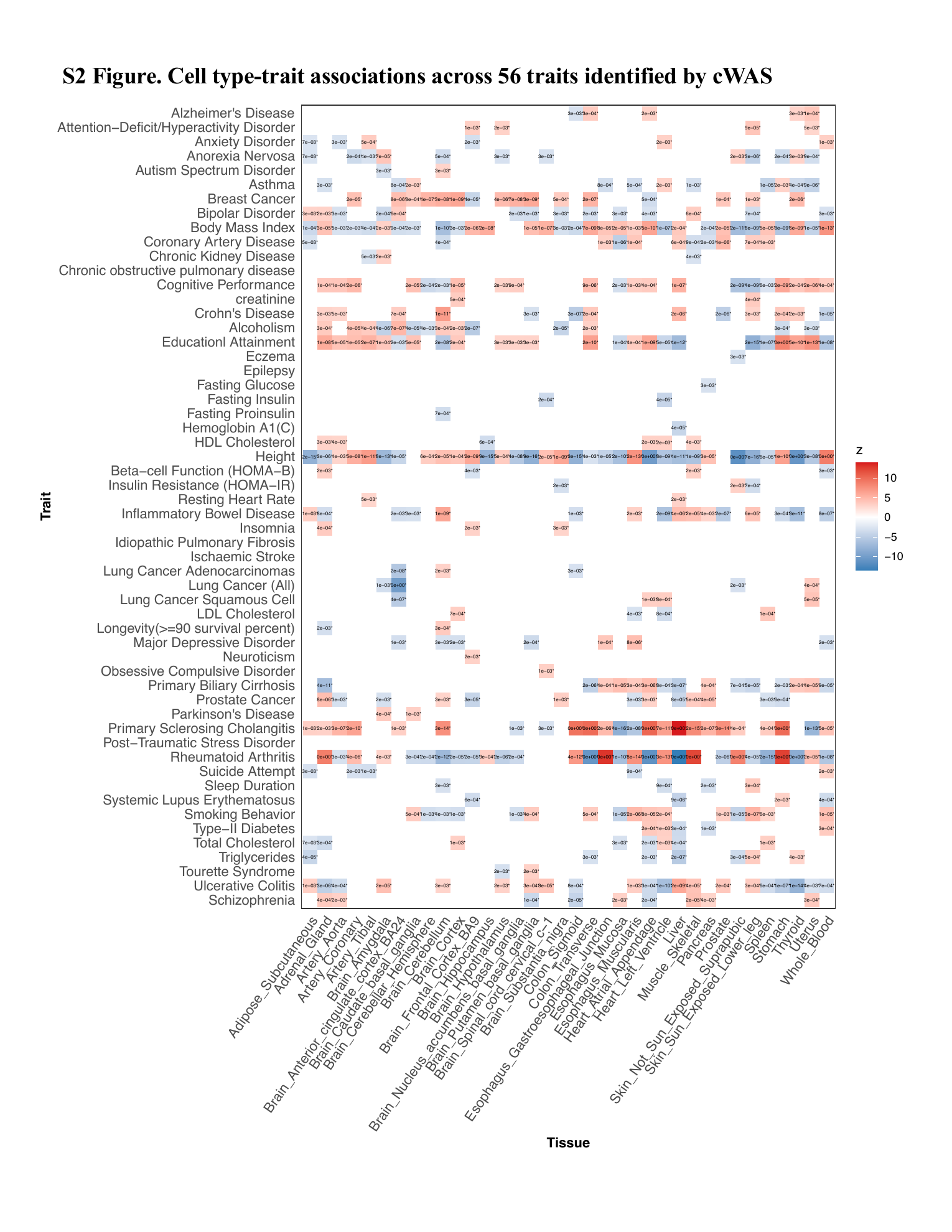

Supplement: S2 Fig — In 36 tissues, the significant/most-significant associated cell type results are shown in the figure. Blue colors indicate the negative correlations between traits and the corresponding associated cell type proportions while red colors indicate the positive correlations. (TIFF) [file pgen.1010825.s002.tiff]

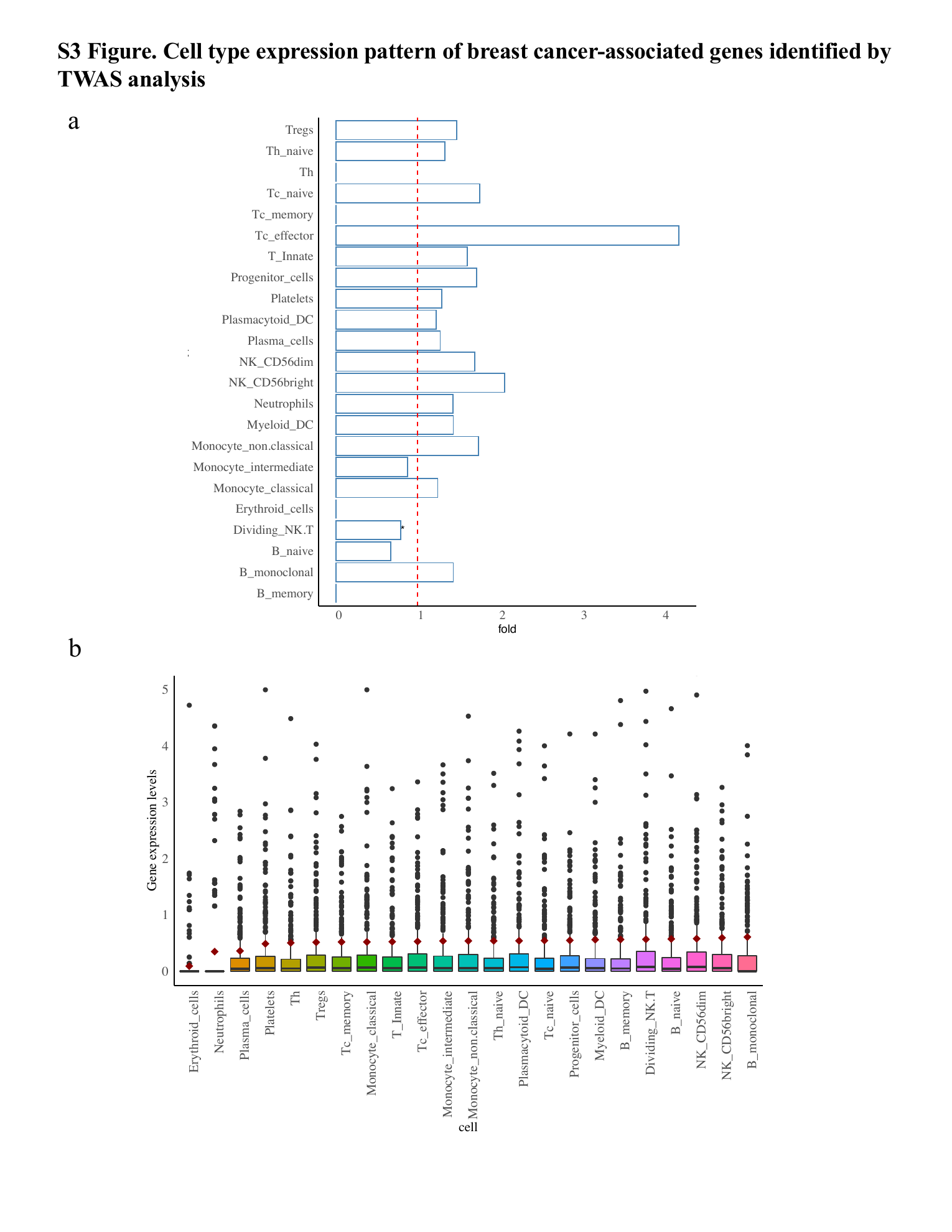

Supplement: S3 Fig — a) As in previous figures, the star indicates the significant cell types after Bonferroni correction in whole blood. The fold indicates (x axis) the enrichment level of breast cancer-associated genes among those genes with high expression specificity in the corresponding cell type. b) Expression levels of identified breast cancer-associated genes in different cell types of whole blood. Red diamonds indicate the mean expression level of breast cancer-associated genes in corresponding cell types. (TIFF) [file pgen.1010825.s003.tiff]

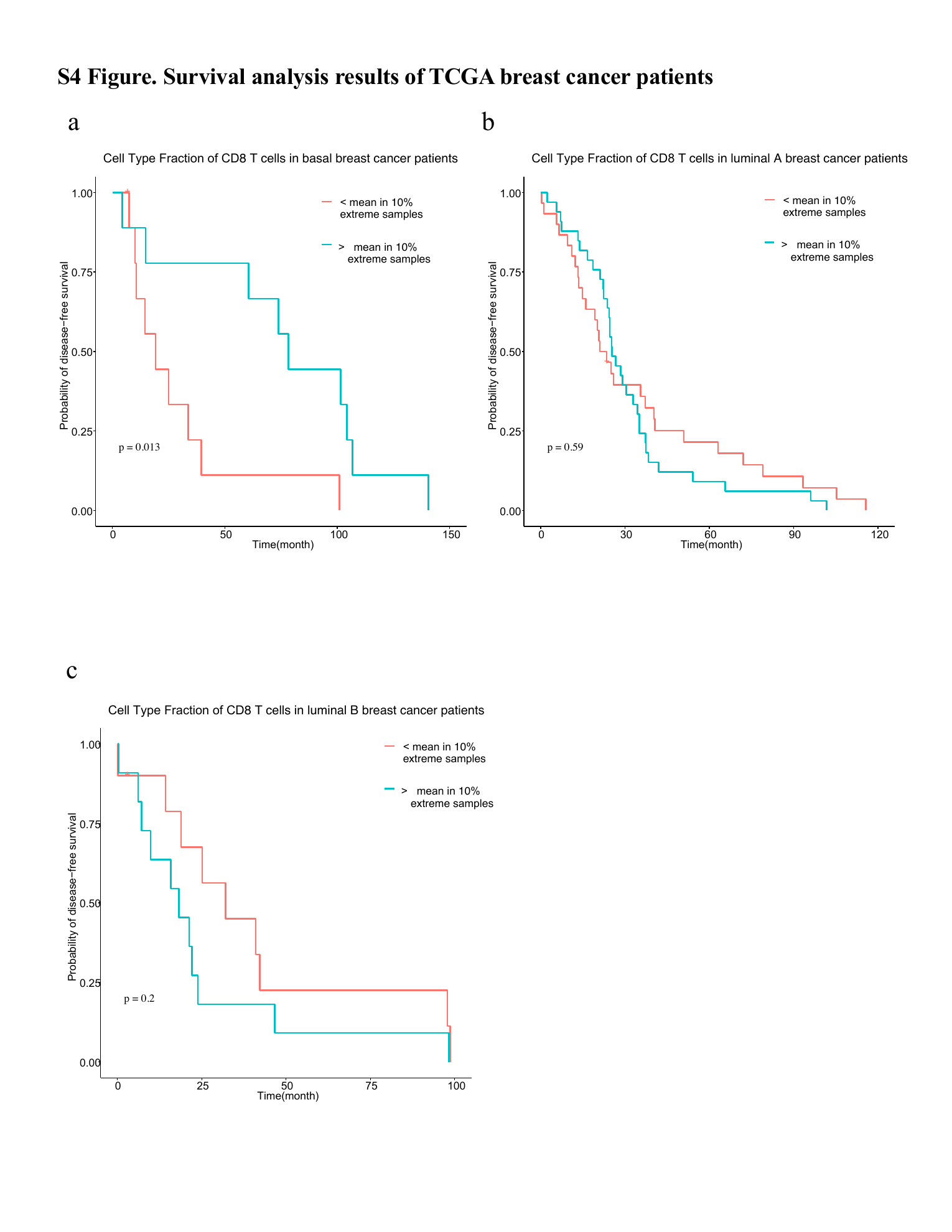

Supplement: S4 Fig — We considered cell type proportions estimated from the assayed expression levels in tumor tissues of TCGA breast cancer patients. a) In patients of European ancestry with basal breast cancer, we compared the survival distributions of patients having the top 10% and bottom 10% of estimated CD8+ T cells proportions. b) For Luminal A patients with European ancestry, we compared patients with the top 10% and bottom 10% of estimated CD8+ T cells proportions. c) For luminal B patients of European ancestry, we compared patients with the top 10% and bottom 10% of estimated CD8+ T cell proportions. We considered different percentages of patients in these comparisons to demonstrate the most significant discriminations between selected two groups of individuals. (TIFF) [file pgen.1010825.s004.tiff]

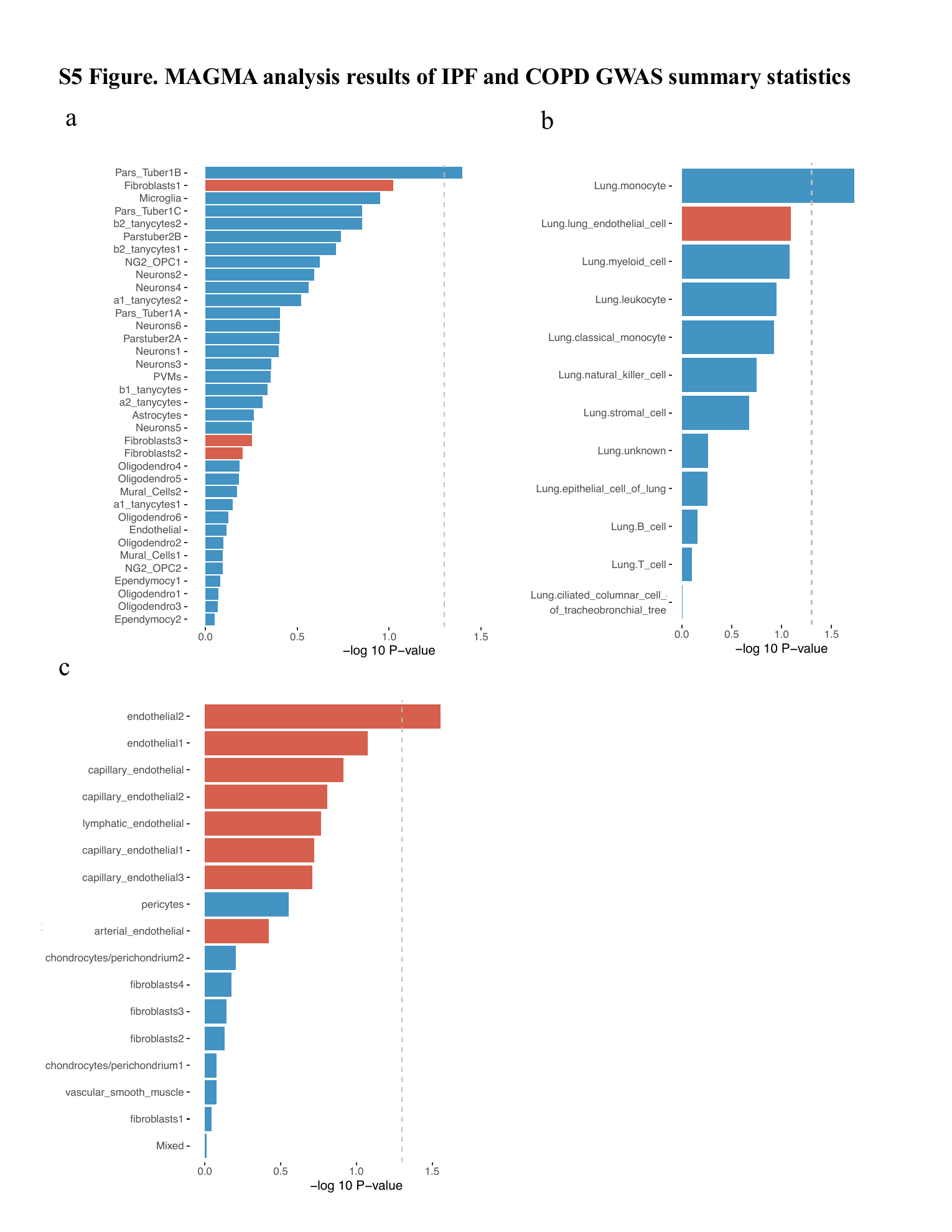

Supplement: S5 Fig — In all figures, the vertical grey dashed lines indicate the significance threshold after Bonferroni correction. The red bars indicate the corresponding cell types of interest in IPF and COPD. a) Bar plots of MAGMA cell type association results between IPF and all cell types of the MAGMA processed GSE93374_Mouse_Arc_ME_level2 dataset [63]. Fibroblast-related cell types are highlighted in red. b) Bar plots of MAGMA cell type association results between COPD and cell types from lung tissue of the MAGMA processed TabulaMuris_FACS_all dataset [64]. Endothelial-related cell types are highlighted in red. c) Bar plots of MAGMA cell type association results between COPD and all cell types from the MAGMA processed GSE99235_Mouse_Lung_Vascular dataset. Endothelial-related cell types are highlighted in red. (TIFF) [file pgen.1010825.s005.tiff]

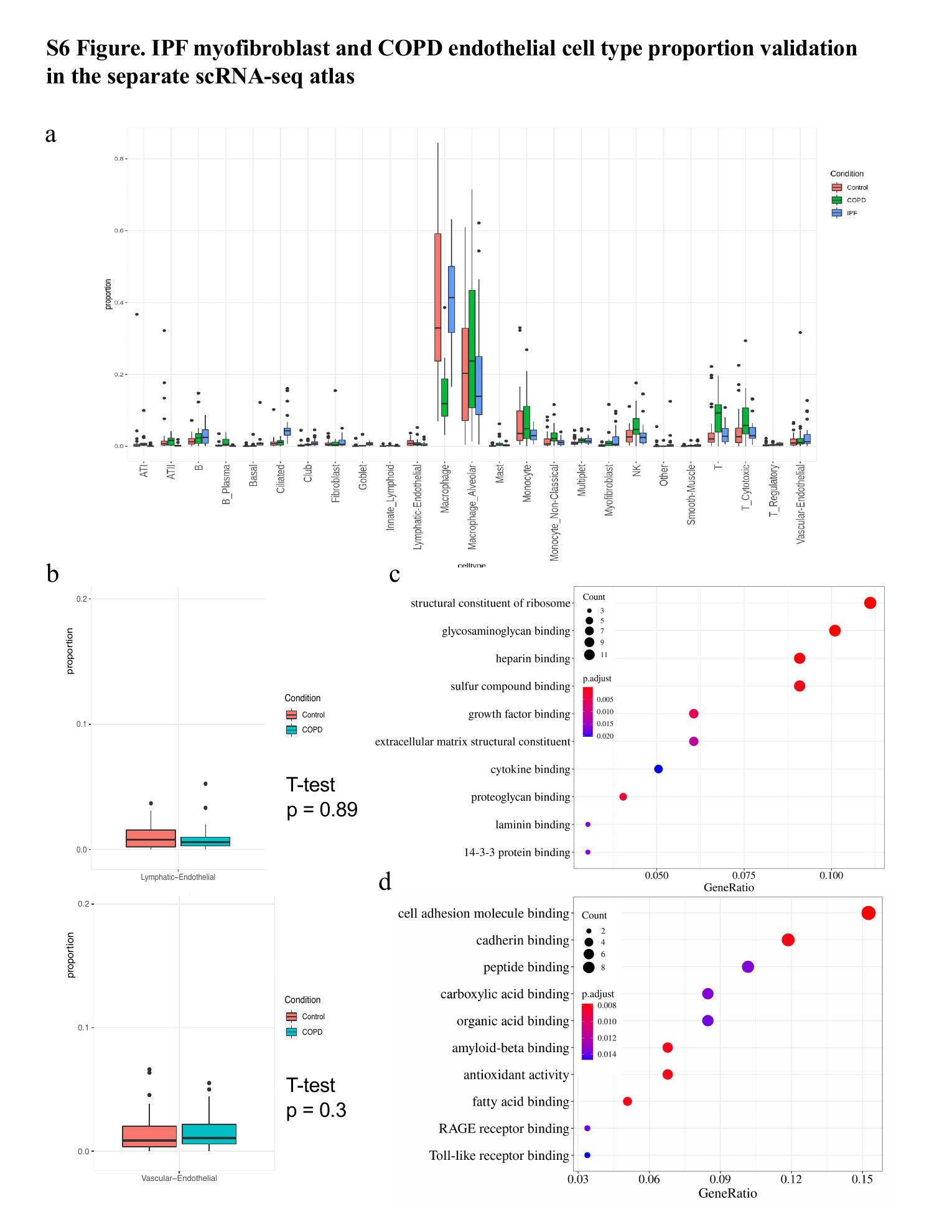

Supplement: S6 Fig — a) Boxplots of cell-type proportions comparisons across IPF, COPD, and controls in lung tissue. The horizontal axis represents the major cell types. The vertical axis is the cell type proportions. The immune cells are the majority of the data. The cell type proportion has a non-negligible variance across different conditions. b) Boxplots of two endothelial subtype proportions comparison between COPD and controls. The vertical axis represents cell-type proportions. To compare the cell type proportion distributions between COPD and controls, we conducted a t-test which was not significant. However, the direction is consistent with cWAS finding for vascular endothelial cells. We still consider these results inconclusive due to the low endothelial cell counts. c) Dot plots of GSEA on IPF myofibroblast down-regulated genes. The dot size is the gene counts found in the pathway. The colors indicate the hypergeometric test p-values. d) Dot plots of GSEA on COPD endothelial down-regulated genes. The dot size and colors are the same as in b). (TIFF) [file pgen.1010825.s006.tiff]

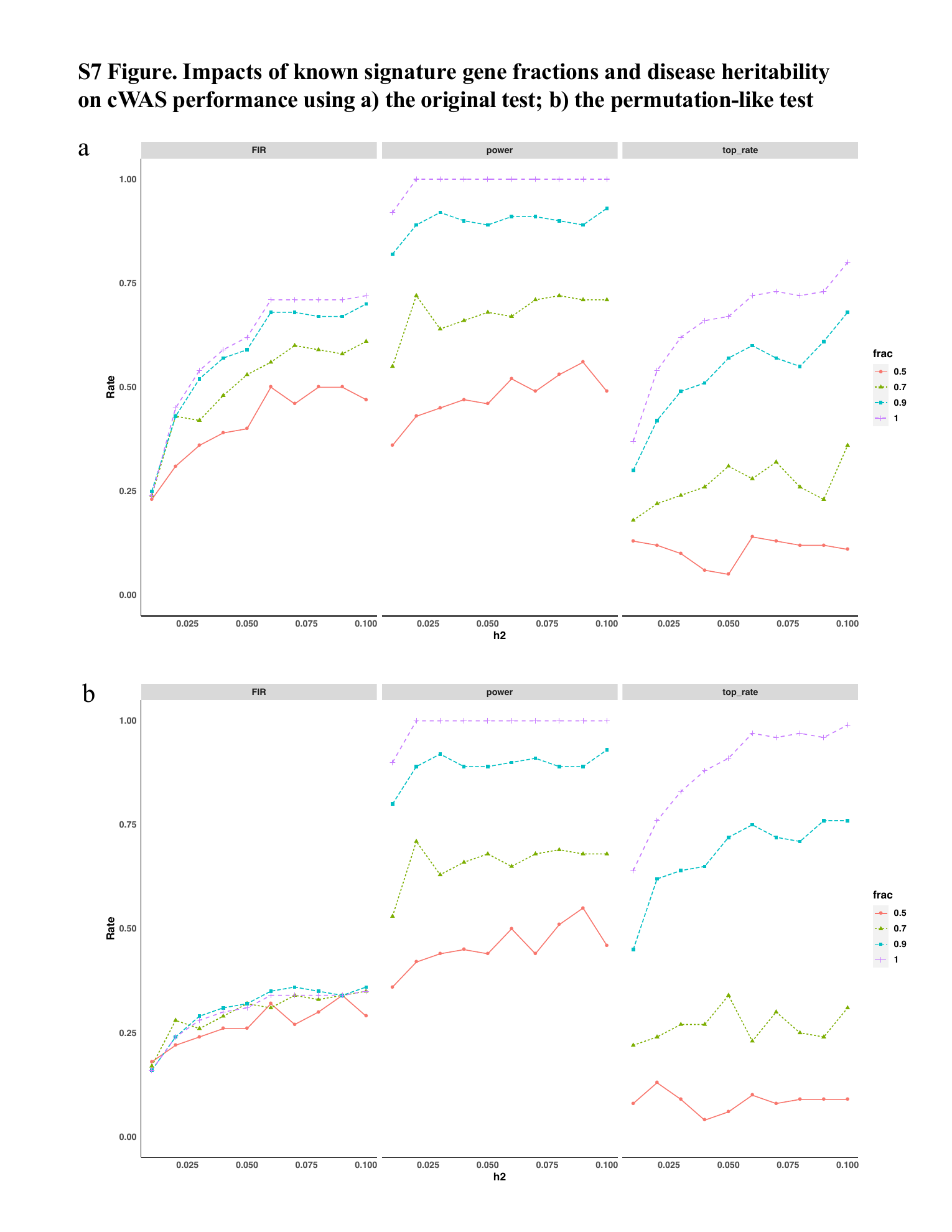

Supplement: S7 Fig — FIR: false identification rate, the rate of cell types which are not disease-associated but identified by cWAS; power: the rate of the true disease-associated cell type being identified as disease-associated; top rate: the rate of the cWAS-identified most significant cell types being the true signal cell type. Note that different from Fig 2, we had 300 replicates for each simulation setting instead of 600 replicates here. a) the results of the original test in the main text; b) the results of the proposed alternative permutation-like test. (TIFF) [file pgen.1010825.s007.tiff]

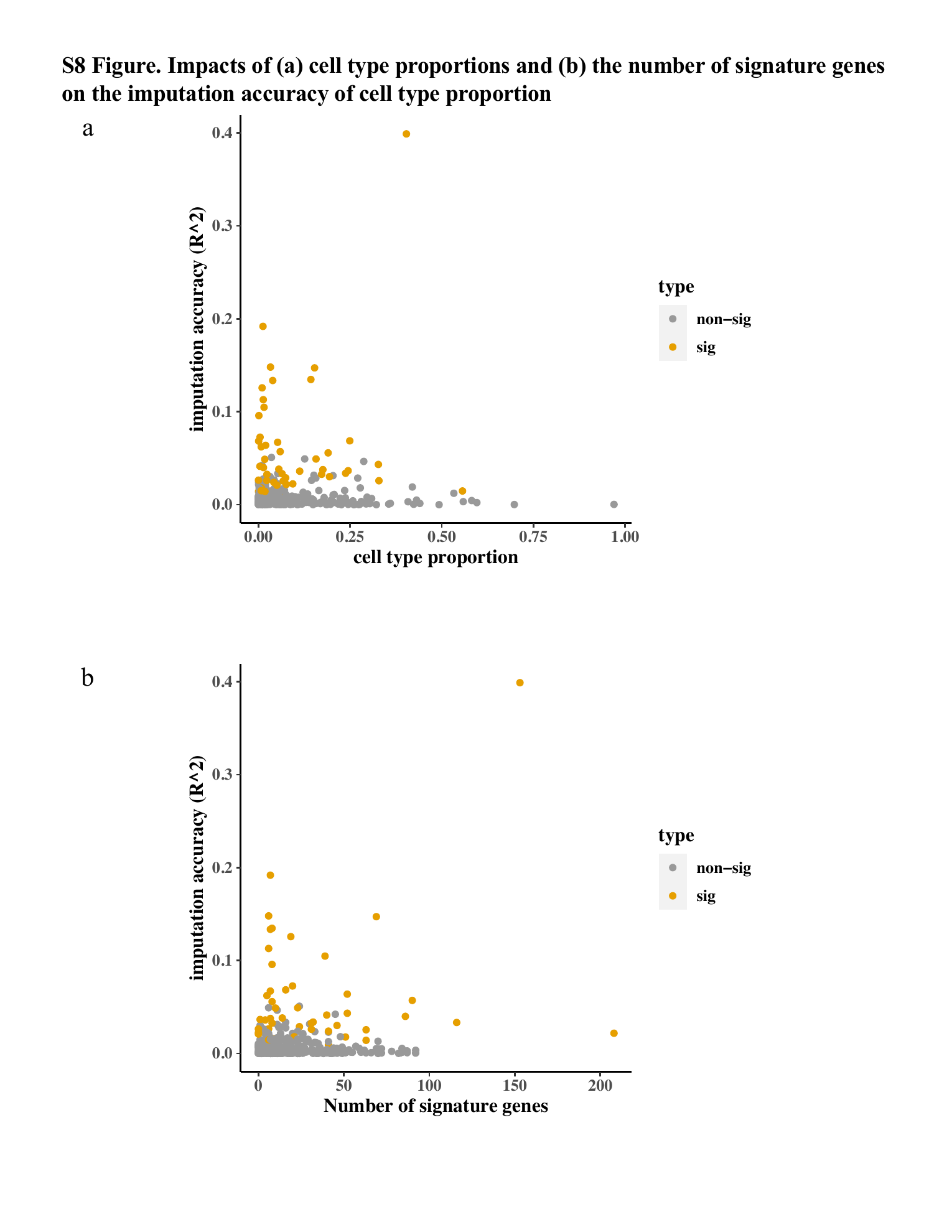

Supplement: S8 Fig — Sig: those cell types whose cell type proportion were imputed with p values <0.05 after multiple test correction. Non-sig: cell types that did not pass the significance level after multiple test correction. (TIFF) [file pgen.1010825.s008.tiff]

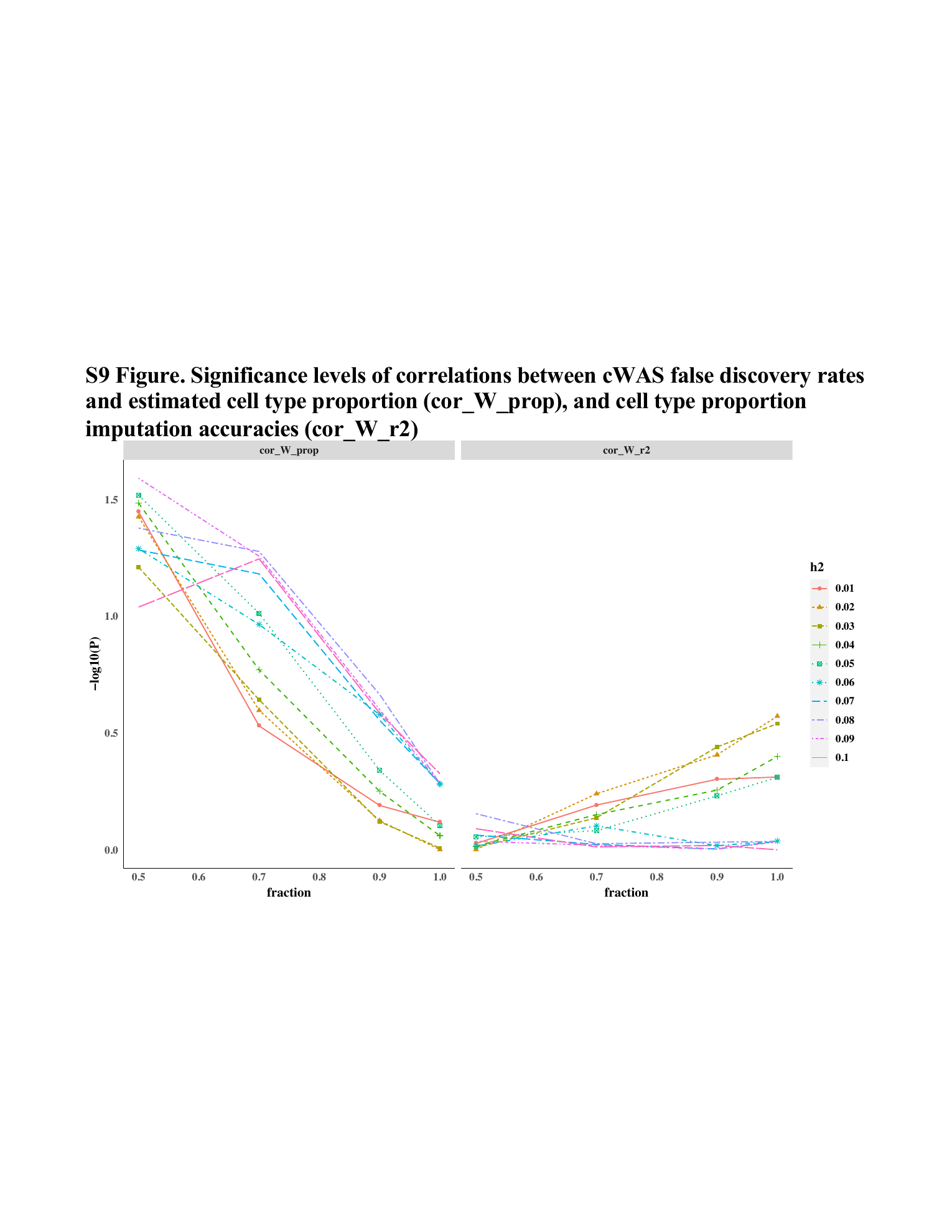

Supplement: S9 Fig — “cor_W_prop” indicates the correlation between the false identification rate of each cell type and its proportion. “cor_W_r2” indicates the correlation between the false identification rate of each cell type and its proportion imputation accuracy. “Fraction” is the fraction of known signature genes, “h2” indicates the percentage of disease phenotype variance explained by genetic-regulated cell type proportions. (TIFF) [file pgen.1010825.s009.tiff]

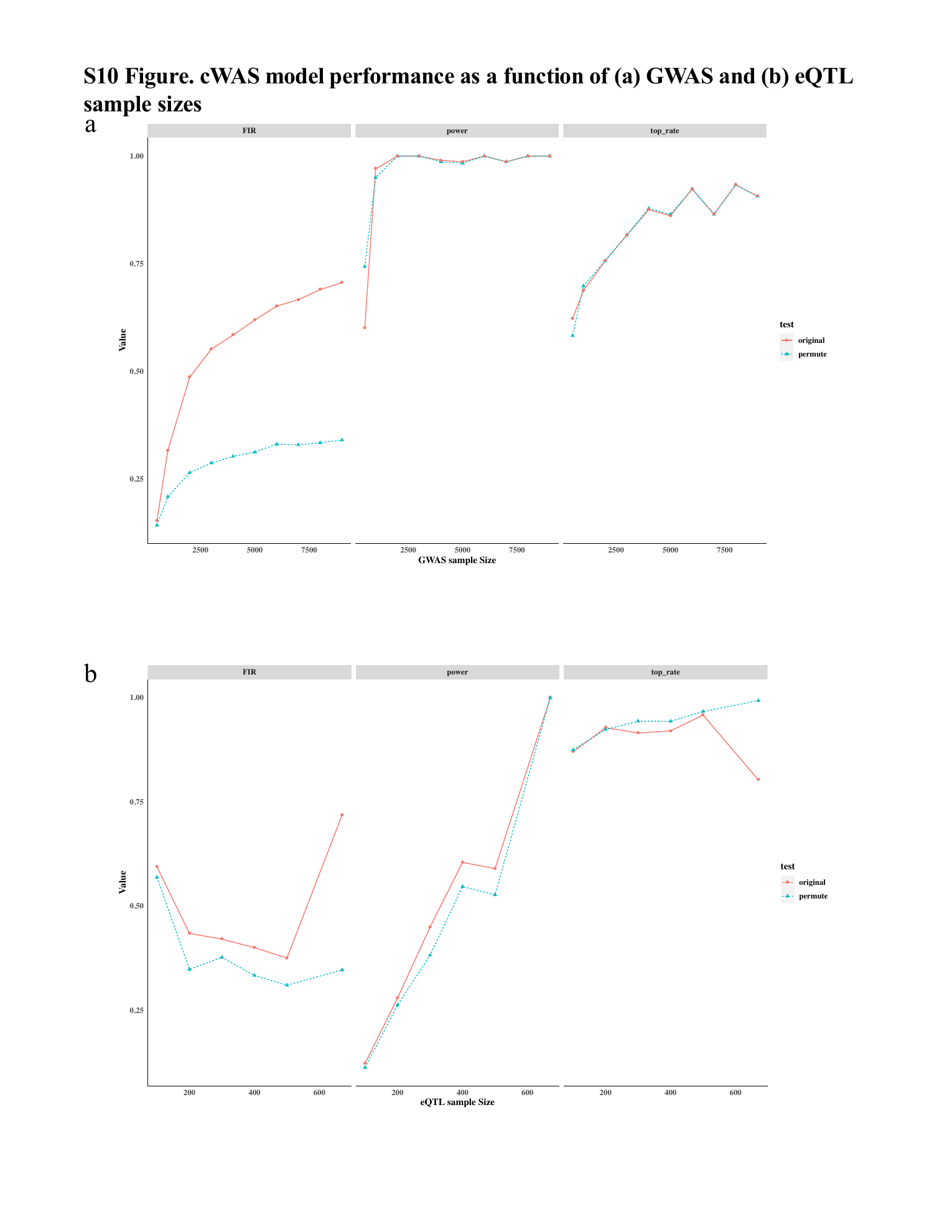

Supplement: S10 Fig — Here we assumed all signature gene are known and the proportion of disease phenotype variance explained by genetic-regulated cell type proportions is 10%. The results of each setting are based on 300 replicates. (TIFF) [file pgen.1010825.s010.tiff]

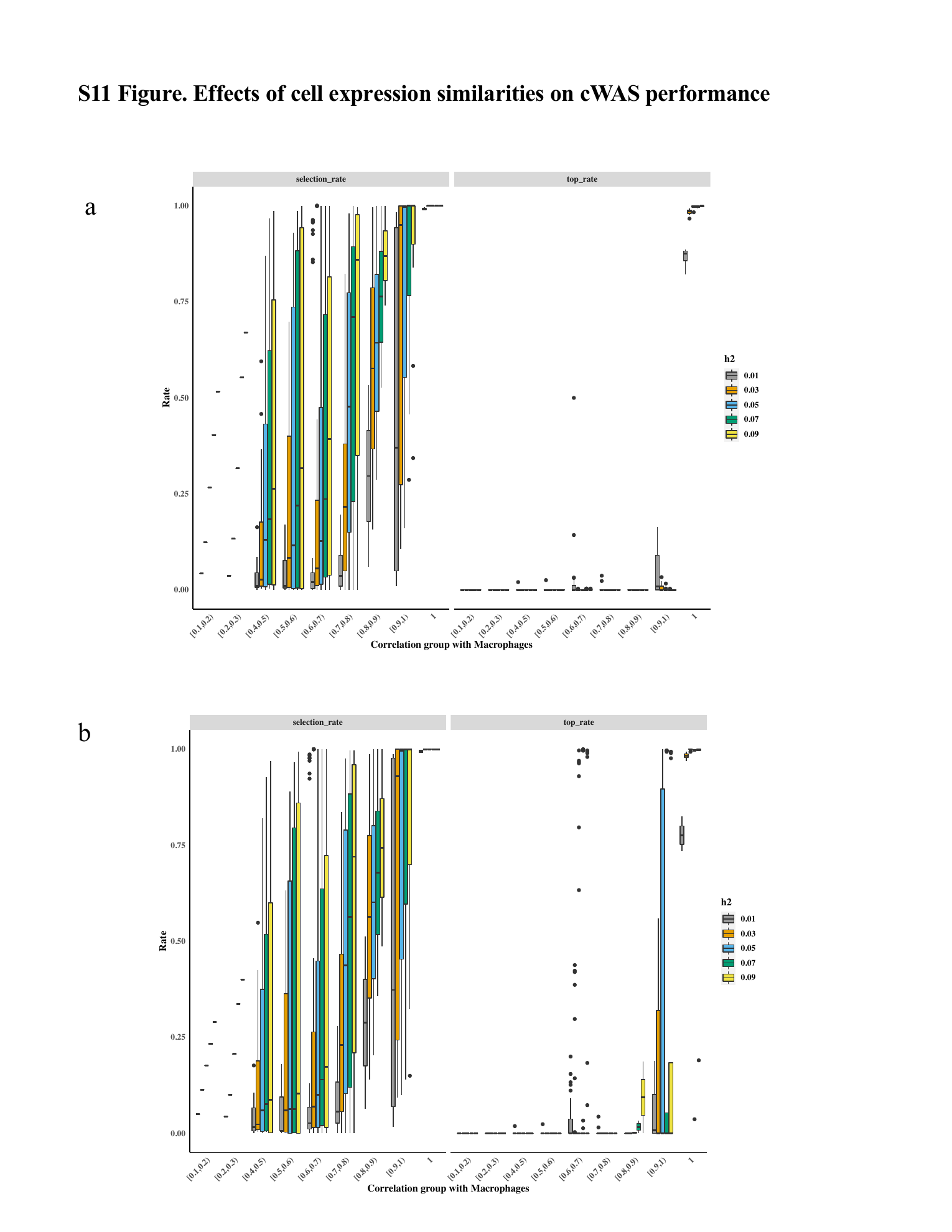

Supplement: S11 Fig — a) The performance of the original test. b) The performance of the alternative permutation-like test. (TIFF) [file pgen.1010825.s011.tiff]

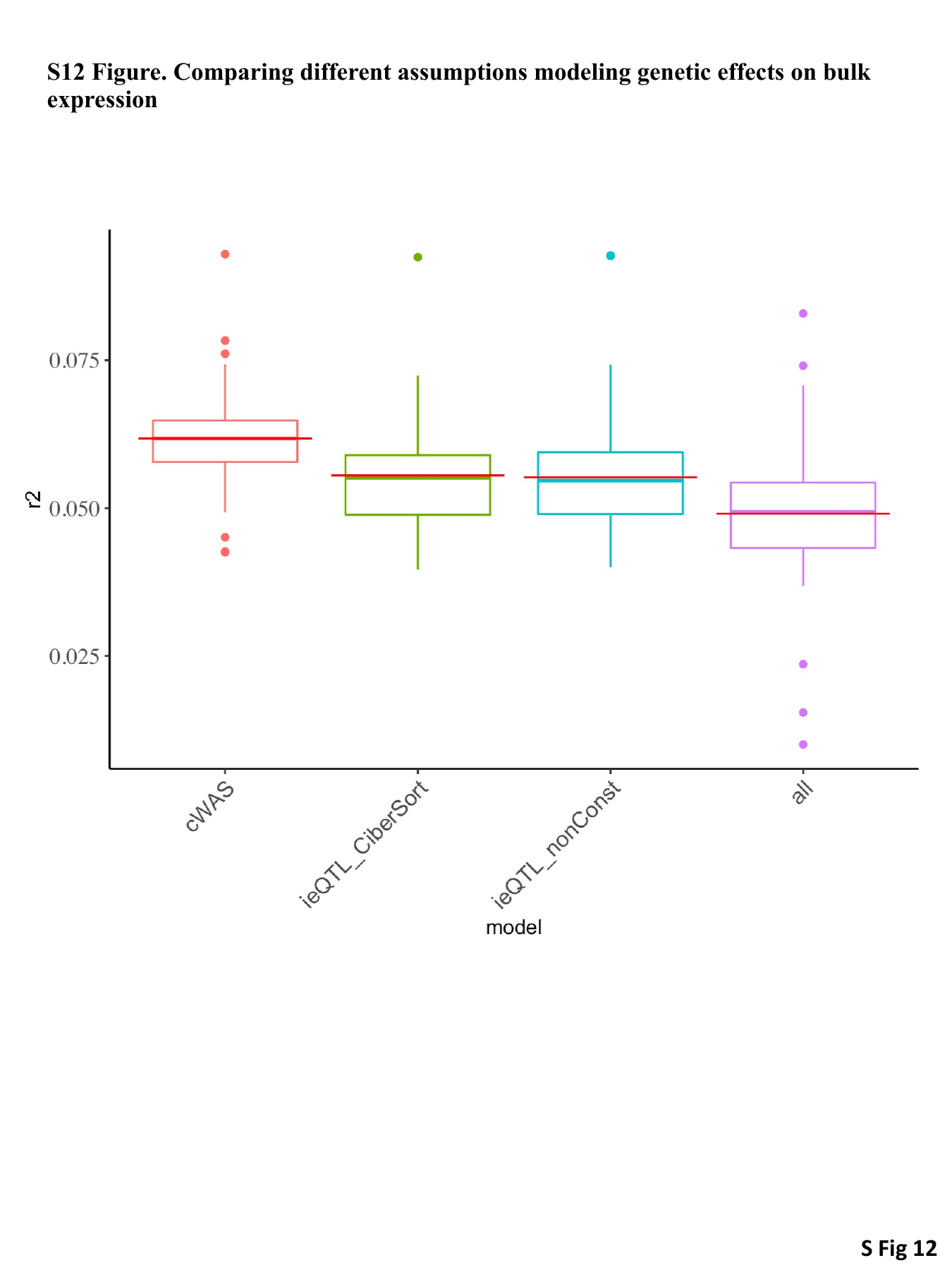

Supplement: S12 Fig — “cWAS” indicates the imputation model under the cWAS assumption. “ieQTL_CiberSort” indicates that the cell-type specific eQTL effect model and the cell type proportion were estimated using CIBERSORT; “ieQTL_nonConst” is similar to the “ieQTL_CiberSort”, but its cell type proportions were estimated using linear regression models without constraints; “all” indicates gene expression modeling considering both cell-type specific eQTL effect and genetic-regulated cell type proportions. (TIFF) [file pgen.1010825.s012.tiff]

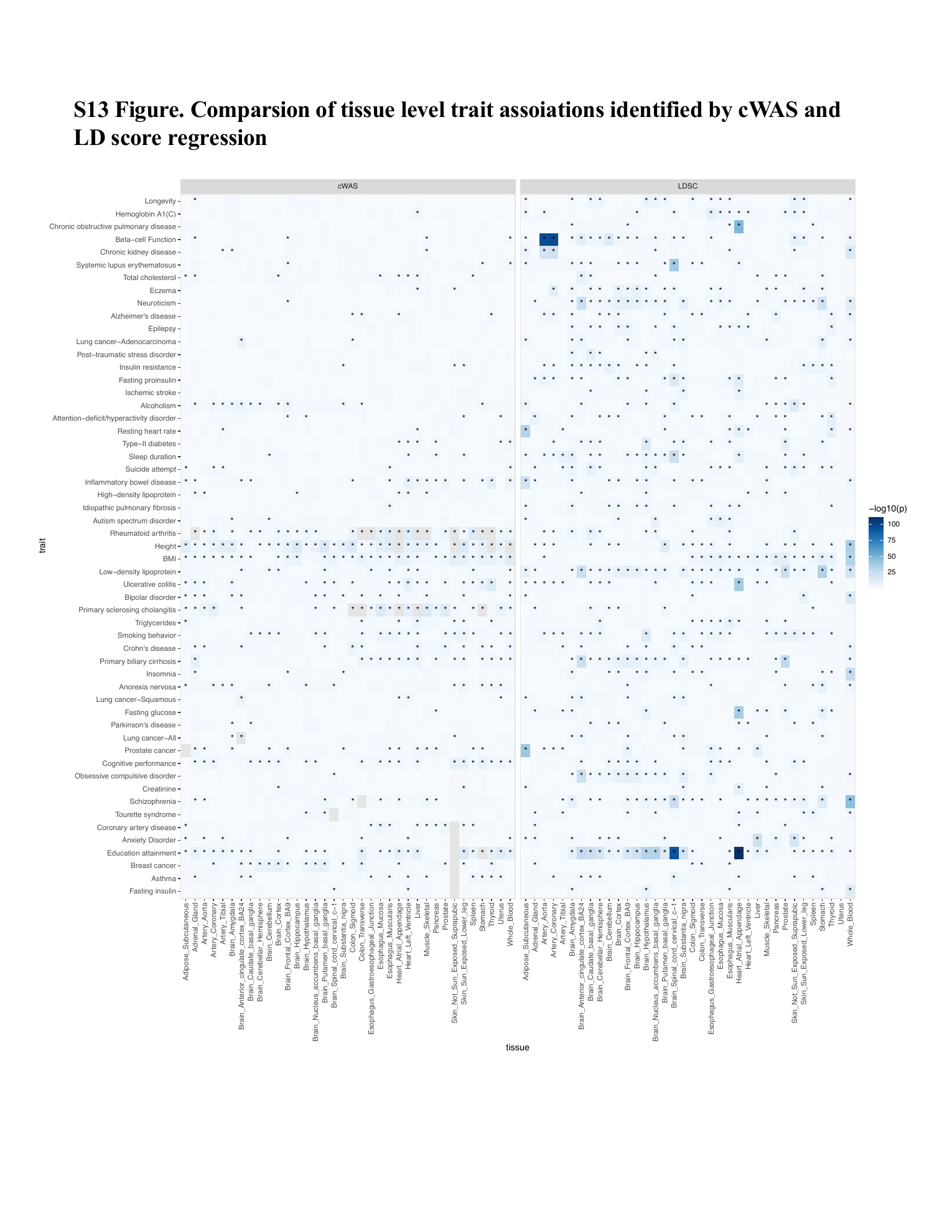

Supplement: S13 Fig — Only traits/tissues with available association results in both methods are plotted. Darker shade represents smaller p-value. Significant associations after Bonferroni correction at alpha = 0.05 are indicated using asterisk. (TIFF) [file pgen.1010825.s013.tiff]

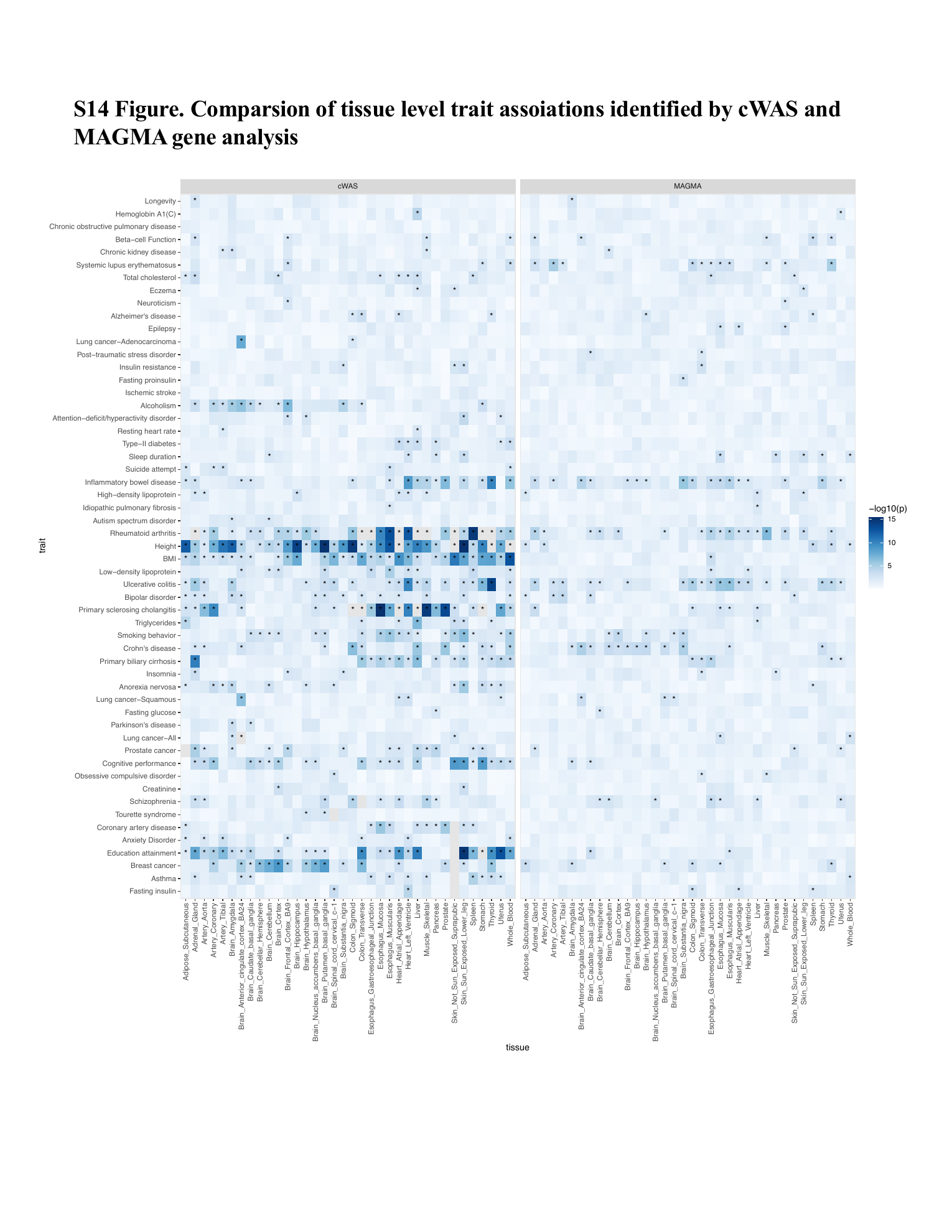

Supplement: S14 Fig — Only traits/tissues with available association results in both methods are plotted. Darker shade represents smaller p-value. Significant associations after Bonferroni correction at alpha = 0.05 are indicated using asterisk. (TIFF) [file pgen.1010825.s014.tiff]

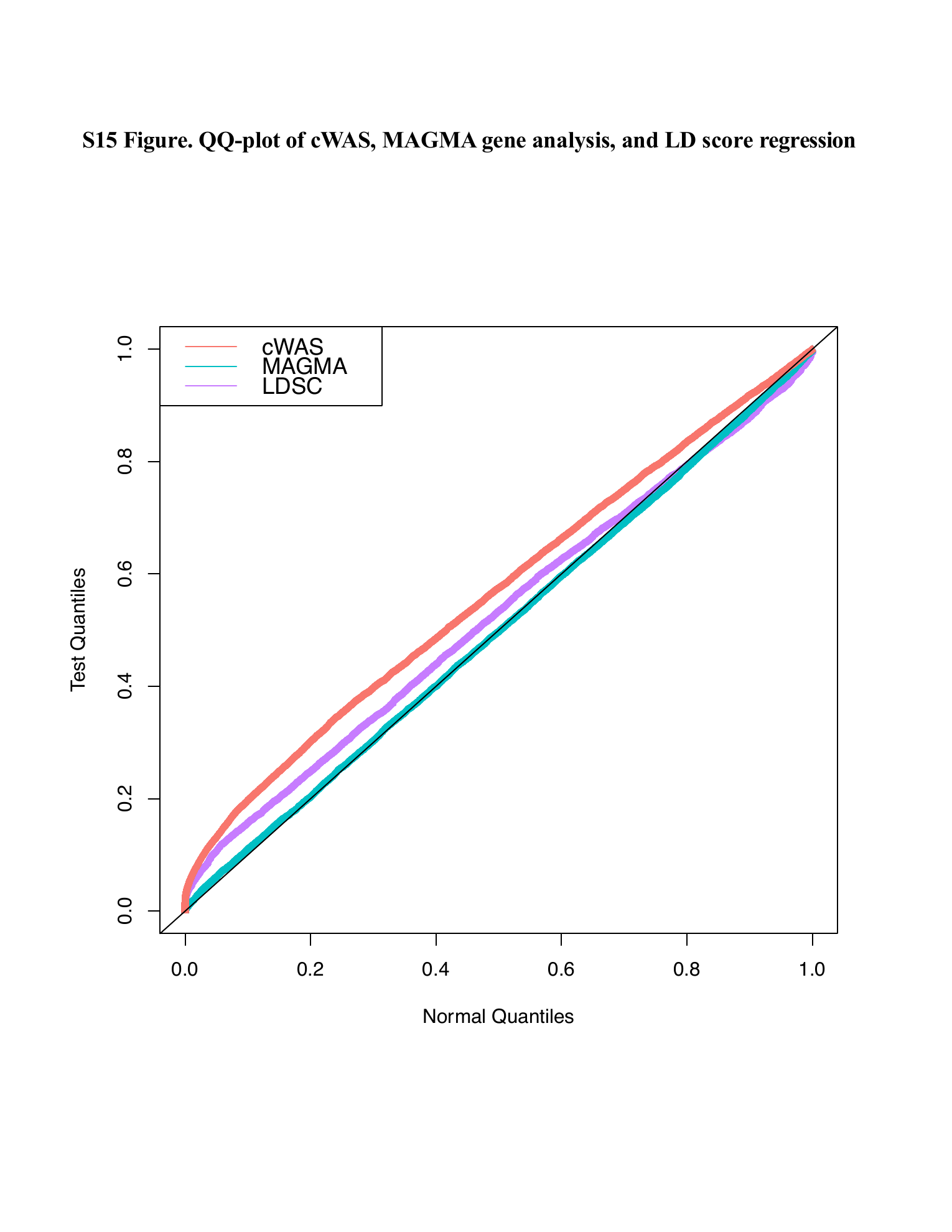

Supplement: S15 Fig — QQ-plot test results for cell type-trait associations across 56 traits. The black line indicates y = x. (TIFF) [file pgen.1010825.s015.tiff]

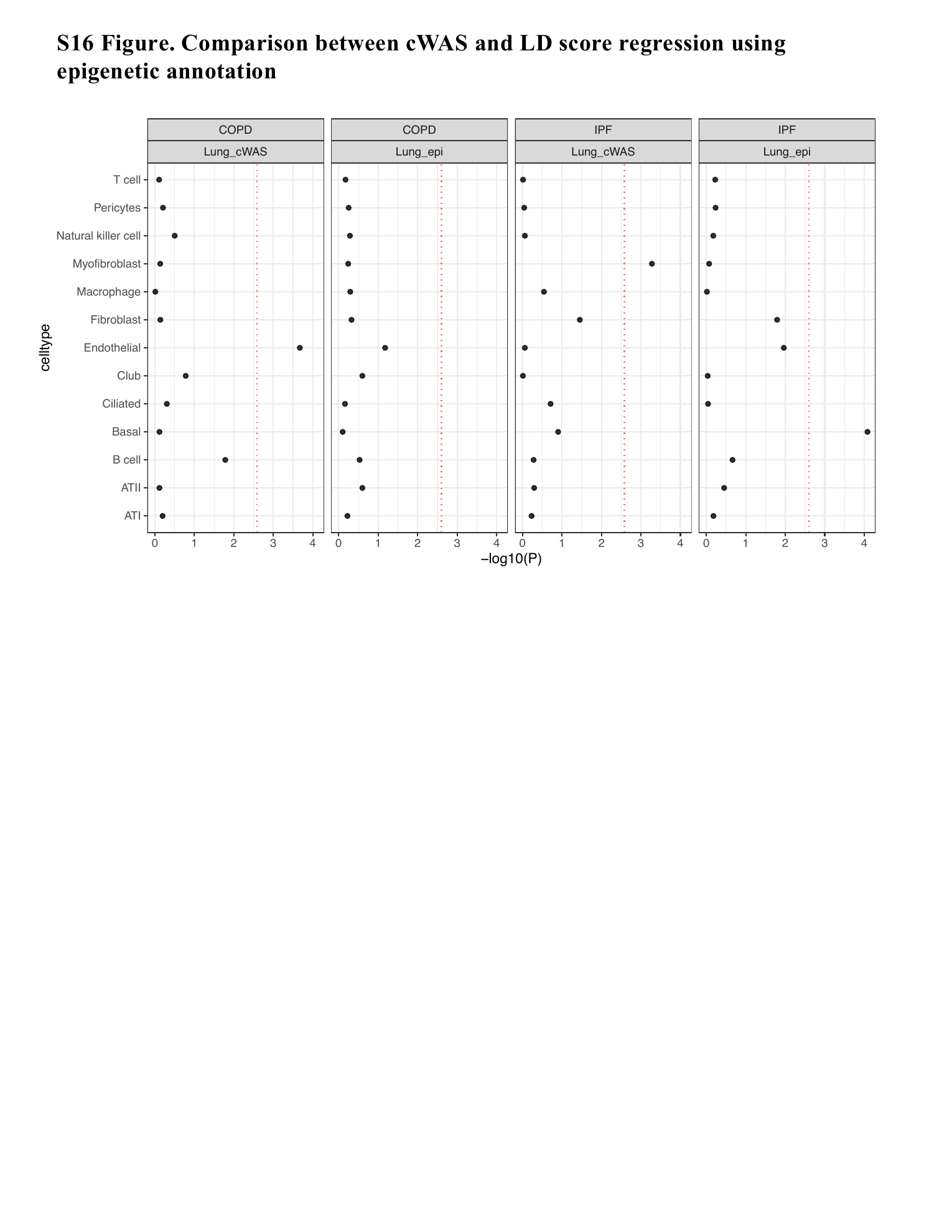

Supplement: S16 Fig — IPF and COPD cell type associations identified by cWAS and LD score regression with epigenetic annotation. Darker shade represents smaller p-value. Significant associations after Bonferroni correction at alpha = 0.05 are indicated using asterisk. (TIFF) [file pgen.1010825.s016.tiff]
