# Supplementary material for: A statistical framework to identify cell types whose genetically regulated proportions are associated with complex diseases
Source: PLoS Genet. 2023 Jul 31;19(7):e1010825. doi: 10.1371/journal.pgen.1010825 (PMC10414598; doi:10.1371/journal.pgen.1010825)
Supplement: S1 Text — (DOCX) [file pgen.1010825.s018.docx]

**False identification rates and an alternative test**

Considering the false identification rate (FIR) as the proportion of cell types which are not associated but identified by cWAS as significantly associated with the simulated phenotype, we evaluated the potential link between the false identification rate, the proportion of disease phenotype variance explained by genetically regulated cell type proportions ($h_{p}^{2}),$ and the proportion of known signature genes $P_{s}$. We observed that the FIR increases as $h_{p}^{2}$ and $P_{s}$ increases (**S7a Fig**). Intrinsically, proportions (genetically regulated or not) of different cell types in the same tissue are highly correlated with each other. When the genetically regulated proportion (GRP) of a cell type has a higher correlation with that of the true signal cell type, the correlated cell type will be more likely to be identified as disease-associated (p=1.8e-2; r=8e-2). To mitigate this situation, we proposed an alternative test - a permutation-like test for the cWAS framework.

We aim to use the test to get the background distribution of the z-statistics quantifying the association between GRPs of cell types and disease phenotypes, while capturing and maintaining the cell type dependency. Specifically, we permuted the GWAS z-scores for 10^6^ times (since the individual data are unavailable) to estimate the mean and standard deviation of the empirical background z distribution (instead of assuming it being a standard z distribution). Then the given p values are used to quantify how significant the difference is between the originally estimated z-statistics and the background distribution. Compared to the original test results (**S7 Fig**, note that in S7 Fig all data were simulated again for making sure the fair comparison between the original test and the new test), the new test has a comparably controlled FIR as 35.9% at most (the corresponding power being 92.9%), while the original test has a higher FIR. On the other hand, the power is comparable between these two tests. The test statistics from these two tests are also significantly correlated (cor:0.88, p<2.2e-16), which further indicates the comparability between these two tests. Also, the statistical power of these two tests is quite similar. We suggest using the permutation-like test when there are multiple disease-associated GRPs identified since its FIR is better controlled based on the simulation analysis while maintain similar statistical power.

Compared to the results presented in Fig 2, the results had a higher variability across different settings. This can be potentially attributed to 1) lower numbers of replicates (300 here compared to 600 in Fig 2) for each setting; 2) lower fractions of known signature genes can potentially lead to higher variability in the signature sampling process, further leading to higher variability across settings.

**Comparisons between cWAS, MAGMA gene analysis, LD score regression, and signal colocalization methods**

To compare cWAS and LD score regression, we used the single-cell datasets from tissues in the Human Cell Landscape (HCL). We performed the Wilcoxon test to identify differentially expressed (DE) genes to make cell-type-specific annotations for signature genes. When a gene is DE for multiple cell types, it was assigned to the cell type with the largest log fold change. After getting cell type annotations, we then investigated cell-type-specific heritability enrichment using LD score regression for 56 traits and 36 tissues.

For MAGMA, we derived tissue-specific signature matrix using the HCL datasets (which were used in cWAS and LD score regression) as customized input. The tissue-specific signature matrix includes average expression levels for each cell types and average expression level for the overall cell population in a scRNA-seq count matrix. MAGMA gene-based analysis was performed using GWAS summary statistics of the 56 traits. Then we conducted the MAGMA gene-property analysis to test for the enrichment of gene-based GWAS signals in expression data in cell types from 36 tissues.

To compare the GWAS colocalization method with cWAS, we used cell counts GWAS of lymphocyte, monocyte, neutrophil, and eosinophil from UK Biobank. We colocalized them with our 56 trait GWAS using the GWAS signal colocalization tool mcoloc by Giambartolomei et al. (2018) on the whole genome. Since mcoloc uses a Bayesian approach, it is difficult to compare with cWAS directly. If we consider the posterior probability of colocalization greater than 0.8 as "significant," then there are no significant results for colocalization.

Since there were no significant findings from signal colocalization, we focused on the comparison between cWAS, LD score regression, and MAGMA results. In total, we investigated 24,192 cell type-tissue-trait pairs (For the 36 tissues from HCL, there were 432 unique cell type-tissue pairs. We tested their associations with each of the 56 traits) using cWAS, LD score regression, and MAGMA. After removing results with invalid output (for example, NA value for p-values), we obtained results of 24,087 cell type-tissue-trait pair using cWAS, 24,162 cell type-tissue-trait pair using LD score regression, and 24,050 cell type-tissue-trait pair using MAGMA.

Overall, cWAS identified much more significant associations than other two methods. cWAS identified 1,055 significant cell type-tissue-trait associations, while LD score regression and MAGMA identified 860 and 239 significant associations, respectively.

Cell types from the same tissue are usually highly correlated with each other. Therefore, comparing the cell type-level results can potentially overestimate the “false positives” of cWAS results. To avoid potential overestimation, we compared the tissue-trait pair identification results instead. cWAS has results available for 2,007 out of 2,016 tissue-trait pairs. And 444 (22.1%) tissue-trait pairs were found to be significant. Around 44.1% (196 out of 444) of tissue-trait pairs identified by cWAS were also identified by LD score regression and/or MAGMA, indicating the high concordance of cWAS results with results from the other two methods.

Many top associations were identified in all three approaches. For example, schizophrenia was associated with multiple cell types in fetal brain (cWAS: smallest p-value = 1.1e-4; MAGMA: smallest p-value = 5.7e-4; LDSC: smallest p-value = 5.0e-25). Height was associated with multiple cell types from whole blood (cWAS: smallest p-value =6.4e-15; MAGMA: smallest p-value = 3.2e-3; LDSC: smallest p-value = 8.4e-14). In addition, the relative strength of associations was similar from different approaches. For example, for ulcerative colitis (UC), cWAS identified stronger association in adult heart (p-value = 1.1e-14) compared to fetal skin (p-value = 3.4e-4). MAGMA also identified stronger adult heart (p-value = 1.5e-7) and weaker fetal skin (p-value = 1.5e-3) associations.

In several scenarios, cWAS identified many associations more biologically meaningful compared to LD score regression. For example, for total cholesterol, cWAS found associations with adrenal gland (p-value= 3.3e-4), heart left ventricle (p-value = 4e-3), heart atrial appendage (p-value=2.1e-3), and adipose subcutaneous (p-value = 7e-3). All of these tissues are biologically closely related to total cholesterol. For comparison, LD score regression found significant associations with brain (p-value =1.1e-9), stomach (p-value = 4.6e-4), pancreas (p-value = 3.1e-3), and prostate (p-value =4.4e-3). Although LD score regression successfully identified pancreas, it also identified stronger association in brain and stomach. It is hard to explain why these two tissues have stronger associations than pancreas.

In addition, we wanted to compare cWAS with a method using epigenetic information as annotation to validate the results from cWAS. For idiopathic pulmonary fibrosis (IPF) and chronic obstructive pulmonary disease (COPD), we applied LD score regression using lung ATAC-seq data to generate cell-type epigenetic annotations. The concordance of cWAS results and LD score epigenetic results was very high (**S16 Fig**). Specifically, fibroblast was found to have moderate significance level using both cWAS (p-value = 3.5e-2) and LD score regression (p-value = 1.6e-2) for IPF. In COPD, cWAS identified endothelial with significant associations (p-value =2.1e-4), and endothelial had the smallest p-value in LD score regression (p-value = 0.06).

**cWAS results on Type-I diabetes**

Utilizing the GWAS summary statistics by D. Calderon et al., we found that the top three cell types in whole blood associated with type I diabetes are naïve B cells (p=0.025), memory B cells (p=0.047), and regulatory T cells (p=0.077). Our finding of regulatory T cells is consistent with previous literature on the important roles of regulatory T cells in type I diabetes. The identified associations between type I diabetes and naïve B cells and memory B cells, which is consistent with a finding of the efficacy of B-cell depletion therapy in patients with type I diabetes by MJ. Smith et al.

**Cell type proportion imputation accuracy**

For tissues used in this study, we firstly estimated their “assayed cell type proportions” using CIBERSORT in each tissue. Then, using the individual genotype data in GTEx, we first imputed their bulk-level genetically regulated gene expression levels (GRE), then estimated their “imputed cell type proportions” by decomposing the bulk-level GRE using unconstrained multiple linear regression (consistent to the cWAS framework). Then we estimated how much of the variation in the “assayed cell type proportions” can be explained by the genetically imputed cell type proportions (**Table 7 in S1 xlsx**). We evaluated the influence of the assayed cell type proportion on the imputation accuracy and did not find significant correlation (p=0.17, cor=0.07, **S8a Fig**). However, the number of specifically expressed signature genes in each cell type was significantly associated with the imputation accuracy in the corresponding cell type (p=4e-4, cor=0.19, **S8b Fig**).

Then we investigated whether the imputation accuracy could affect the final cWAS test results. In real data analysis, we found that there was no significant correlation between imputation accuracy and the rate of the corresponding cell type being selected as disease-associated (p=0.31, cor=-0.09). In simulation analysis only using whole blood, we found similar result with no significant correlation between the FIR and the fraction of genetically explainable proportion variance across cell types (p=0.99, cor=0.03), when the GRP of cell type explained 10% of disease phenotype variances. However, we did observe an interesting but not-surprising pattern (**S9 Fig**) that as the fraction of known signature genes increased, the correlation between FIR and assayed proportion level became less significant (**S9a Fig**). Here, we model the correlations using the linear model FIR$\sim$ assayed proportion level $+$ fraction of genetically explainable cell type proportion variance. Also, when heritability was larger, the correlation between FIR and assayed proportion levels was comparably more significant while the correlation between FIR and the fraction of genetically explainable proportion variance was less significant.

**Sample sizes and cWAS model performance**

Using the whole blood tissue (n=670) as the eQTL study and 10,000 samples from UK biobank, we simulated disease phenotypes based on GRP of M1 Macrophages and evaluated how power, top selection rate (the fraction of the most significantly associated cell types being the true signal cell type), and FIR are affected by the sample sizes of eQTL and GWAS studies (**S10 Fig**). Here we assumed that the GRPs of M1 Macrophages explained 10% of disease phenotype variance and all signature genes were known. We investigated the effects of sample sizes on cWAS performance using the model: metric $\sim$ GWAS sample size $+$ eQTL sample size.

Compared to GWAS sample size (p=0.16), the eQTL sample size (p=3.6e-7) has more significant effects on the statistical power. This indicates that the imputation weights/models for bulk-level gene expression are more likely to limit the statistical power of cWAS models. Both the original test and the permutation-like test showed similar patterns. The top selection rate was more likely to be affected by GWAS sample size (p=9.7e-4) than the eQTL sample size (p=0.47). More importantly, for both the original test and the permutation-like test, when the sample size of eQTL studies was smaller than half of the sufficient sample size (n=670 in our simulation), the statistical power was even smaller than the corresponding FIR, indicating the important role of eQTL sample sizes in cWAS analysis.

Other factors, such as the contribution of cell type GRP to disease phenotypes and gene expression heritability, can also affect cWAS performance. By directly looking into different sample sizes of GWAS and eQTL studies, we found that more eQTL studies with larger sample sizes will be needed to identify disease associated GRPs with a higher statistical power and lower FIR. Also, for tissues with comparably small sample sizes, we suggest more cautions for the interpretation of cWAS results.

**The effects of cell expression similarities on cWAS performance**

As the previous sections showed, highly dependent/correlated cell types can potentially be identified simultaneously as disease-associated by cWAS. Here we systematically investigated how cell similarities can affect cWAS performance. We only considered the signature gene expression patterns across cell types and used the expression similarities of those signature genes to represent cell similarities across cell types. We used single cell data from the lung tissue instead of the whole blood tissue (which was used in other simulation analysis of this work) since we have comparably higher resolution than single cell data in lung tissue. We simulated disease phenotypes with GRPs of cell types using signature matrix with different cell-cell similarities. Disease phenotypes were simulated based on the GRPs of macrophage in lung tissue, with varying percentages of phenotype variances explained by its GRPs ($h_{p}^{2}$).

We grouped cells based on their expression correlation (of signature genes, the absolute value) with macrophage into 10 categories. With $h_{p}^{2}$ increasing, the probability of a disease non-associated cell type being selected (defined as the selection rate) also increased (cor: 0.32, p<2.2e-16, **S11 Fig**). Also, the selection rate was positively associated (cor: 0.48, p<2.2e-16) with this cell type’s expression correlation (the absolute value) with the true signal cell type (macrophage). More specifically, when the correlation was larger than 0.9 and $h_{p}^{2}\geq0.03$, the selection rate was higher than 0.72, which makes the identification of the true disease-associated cell type difficult. However, if we look at the top selection rate (the rate of each cell type selected as the most significant cell type), the true disease-associated cell type would always have the highest top selection rate, significantly higher than any other cell types. Therefore, we suggest either focusing on the most significant disease-associated cell types identified by cWAS or interpret the results with caution when multiple highly correlated cell types are identified by cWAS. Similar patterns were also observed in the results of the permutation-like test (**S11b Fig** ).

**Differences of trait correlations between fetal and adult tissues**

Based on cWAS-identified signals in 13 fetal brain tissues and 21 adult non-brain tissues, we estimated trait-trait correlations respectively in fetal and adult tissues. In fetal tissues, 122 significant trait-trait correlations were identified while 119 significant ones were identified in adult tissues. 24 trait pairs showed significant correlations in both fetal brain tissues and adult non-brain tissues. 2 out of those 24 pairs showed correlations of different directions – Asthma - smoking initiation (SmkInit) pair and cognitive performance (CP) - type 2 diabetes (T2D) pair. Asthma and SmkInit are positively correlated in fetal brain tissues (cor=0.35, p=1.13e-6) while showed negative correlation in adult non-brain tissues (cor=-0.33, p=1.24e-7).

**Comparisons of different modeling assumptions for genetic effects on bulk expression**

We assumed that the cell-type specific genetic effects on gene expression are mediated by regulating cell type proportion(s). Recent GTEx studies also investigated the cell-type eQTL effects by assuming that genotypes may have different effects regulating gene expression levels in different cell types. More specifically, we can consider the following two models:

$$M_{0}:B_{n\times a}=F_{n\times c}^{g}S_{a\times c}^{T}=G_{n\times p}\gamma_{p\times c}S_{a\times c}^{T};F_{n\times c}^{g}\propto G$$

$$M_{1}: B_{n\times1}^{i}={diag(F}_{n\times c}{(G}_{n\times p}\beta_{p\times c}^{i})')$$

where $n$ is the number of individuals, $a$ is the number of genes, $c$ is the number of cell types, $B$ is the bulk-level gene expression matrix, $F^{g}$ is the cell type proportion matrix regulated by genetics, and $S$ is the cell-type specific gene expression level, which is the same across individuals. $G$ is the genotype matrix. $F$ is the assayed cell type proportion. $\gamma$ is the genetic effect vector on cell type proportions while $\beta$ is the genetic effect vector on cell type-specific expressions. $diag(A)$ is taking the diagonal vector of any matrix $A$. Under model $M_{0}$, which is the model used in cWAS framework, we assume that all individuals have similar signature gene expression levels but different cell type proportions. Therefore, under model $M_{0}$, the main individual bulk expression level differences come from different cell type proportions, which are regulated by genetics. Model $M_{1}$ assumes that both signature gene expression levels in each cell type and cell type proportions vary across individuals. Under $M_{1}$, genetics only regulate gene expression levels in each cell type. For model $M_{1}$, for simplicity, here we are only showing the relationship between genetics and the bulk gene expression level for any single gene $i$.

To compare these two models, we 1) first compared the imputation accuracy of these two types of models using elastic net models with 5-fold cross validation; and 2) investigate the trans-eQTL effects of SNPs used for imputing cell type proportion.

To model the cell-type specific eQTL effects in $M_{1}$, we performed interaction analysis as that in GTEx analysis as $B\sim G+G\circ F_{.1}+G\circ F_{2}+\ldots+G\circ F_{c},$ where $F_{i}$ is the assayed cell type proportion for cell type i. However, instead of using the cell type enrichment scores, we used the CIBERSORT-inferred cell type proportions (and cell type proportions inferred by linear regressions without considering non-negative or sum constraints, for consistency with cWAS model). To attain the best imputation performance for both models and avoid potential over-fitting problems, we applied 5-fold elastic net models to estimate the imputation accuracy for both models. For model $M_{0}$, we modeled gene expression as $B= \hat{F}\gamma$ where  $\hat{F}\sim G\hat{T}$ is genetic-imputed cell type proportion matrix, $\gamma$ is the regressed cell-type level gene expression and $\hat{T}$ is regressed coefficients when imputing cell type proportions using genotype information. We used the squared correlation ($R^{2}$) between the imputed gene expression and assayed bulk expression from GTEx in 5-fold cross validation to evaluate the performance of these two models. Compared to the cell-type-specific eQTL model ($M_{1}$), the proportion-QTL model ($M_{0}$) achieved statistically significantly better performance across tissues (p=0.02 compared to models using CIBERSORT-inferred proportions, p=0.01 compared to models using linear regression-inferred proportions, S12 Fig). When jointly modeling $M_{0}$ and $M_{1}$ effects, we observed even worse model performances compared to cWAS (p=1e-4, S12 Fig), which suggests over-fitting due to insufficient sample sizes in the GTEx data.

Also, we evaluated the trans-eQTL effects of SNPs used in the imputation models of signature genes. Due to limited sample size in GTEx, it is hard to recall the significant transeQTL-eGene pairs (163 pairs in total across all tissues) discovered in GTEx study (v8). Instead, we compared the trans-eQTL signals of those cis-eQTLs for signature genes to the trans-eQTL effects of all SNPs across the whole genome. Since GTEx only provides the significant trans-eQTL-eGene pairs, we do not have the background trans-eQTL effects for comparison. As a compromise, we assumed the whole genome p-value distribution of the trans-eQTL effects as the uniform distribution. Using the Kolmogorov–Smirnov test, we found that the p value distribution of trans-eQTL effects of cWAS SNPs was significantly different from the whole genome/background distribution (p<2e-16) across all 36 tissues.

Alternatively, we considered that if the true genetic effects can be mediated by both regulating cell-type specific gene expression (cell-type specific eQTLs) and affecting cell type proportions (proportion QTLs), we found that our model is more like to capture the component mediated by proportion QTLs if we assume these two effects are independent of each other as follows:

$$G_{n\times p}\eta_{p\times1}=\hat{B}_{i}=\hat{B}_{Fi}+\hat{B}_{si}=G_{n\times p}\gamma_{p\times c}S_{i\times c}^{T}+ diag(F_{n\times c}{(G}_{n\times p}\beta_{p\times c}^{i})')$$

where $\eta$ is the overall eQTL effects as defined in GTEx study and gene expression imputation models, and $\hat{B}$ is the genetic regulated expression and we assumed a linear additive model here. $G_{n\times p}\gamma_{p\times c}S_{i\times c}^{T}$stands for gene expression only affected by genetic-regulated cell type proportion for gene $i$, $diag(F_{n\times c}{(G}_{n\times p}\beta_{p\times c}^{i})')$ stands for the other part of gene expression only affected by genetic-regulated cell-type specific gene expression for gene $i$. $\gamma$ is the genetic effect on cell type proportions and $S$ is the signature gene expression. It is reasonable to assume the independency between two components $\hat{B}_{F}$ (the part affected by genetic-regulated proportion) and $\hat{B}_{s}$(the part affected by genetic-regulated cell type-level expression) since $S_{1\times c}^{T}$ and $F_{n\times c}{(\beta}_{p\times c}^{i})'$ should be independent on each other (one capturing the gene expression level shared across individuals and the other capturing gene expression levels unique to each individual), therefore by simply projecting overall eQTL effects $\beta$ on the cell-type specific gene expression matrix $S$, we can potentially get the bulk-level eQTL effects mediated by regulating cell type proportions, which is basically what cWAS model does. Therefore, even under this alternative assumptions, cWAS model should still hold.

The proof of the independency between model $M_{0}$ and $M_{1}$ can be seen as follows:

$$cov\left( XC,YZ \right)=E\left( C^{T}X^{T}YZ \right)-E\left( XC \right)^{T}E\left( YZ \right)$$

$$=C^{T}E\left( X^{T}YZ \right)-C^{T}E\left( X^{T} \right)E\left( YZ \right)$$

$$=C^{T}[E\left( X^{T}YZ \right)-E\left( X^{T} \right)E\left( YZ \right)]$$

$$=C^{T}cov\left( X^{T},YZ \right)$$

$$=0$$

where $C$stands for the signature gene matrix in M0, which is a constant matrix. $X$ is the genetic-regulated cell type proportion matrix in M0. $Y$ is the assayed cell type proportion matrix and $Z$ is the genetic-regulated cell-type level gene expression matrix in M1. Based on our assumption, $X$ is independent from $YZ$, which leads to their covariance being 0, indicating M0 and M1 should be independent from each other.

We considered the bulk-level gene expression imputation models only for signature genes to get genetic-effects mediated by regulating cell type proportions here,

$$B_{n\times g}=X_{n\times p}\beta_{p\times g}+T_{n\times g}$$

$$B_{n\times g}=\left( \begin{matrix} F_{1\times c}^{1}E_{c\times g}^{1} \\ \vdots\\ F_{1\times c}^{n}E_{c\times g}^{n} \end{matrix} \right)$$

where $B_{n\times g}$ is the bulk gene expression matrix for $n$ samples and $g$ genes in a tissue, $X_{n\times p}$ is the genotype matrix of $n$ samples and $p$ SNPs, $\beta$ is the eQTL effect matrix, $T_{n\times g}$ is the matrix capturing non-genetic effects, $F_{n\times c}$ is the true cell type proportion matrix, and $E_{c\times g}^{i}$ is the cell-type-level expression matrix of $g$ genes in $c$ cell types for sample $i$. The top equation includes the genetically-regulated part and non-genetic part of bulk-level gene expression when modeling bulk-level gene expression. The second equation quantifies the relationship between bulk-level and cell-type-level gene expression.

For the signature genes used in cWAS models, their cell-type-level expression matrix $E$ is comparably similar/fixed across samples, which means that $E_{c\times g}^{1}\approx E_{c\times g}^{2}\approx\ldots\approx E_{c\times g}^{n}\approx S_{c\times g},$where $S_{c\times g}$ is the cell-type-level signature matrix which helps to signify different cell types. That means that cell-type-specific expression levels of those signature genes are similar across different genotypes. Then bulk expression level variances of those signature genes are more likely due to variances of cell type proportions, where we have

$$B_{n\times g}=X_{n\times p}\beta_{p\times g}+T_{n\times g}$$

$$B_{n\times g}=\left( \begin{matrix} F_{1\times c}^{1}E_{c\times g}^{1} \\ \vdots\\ F_{1\times c}^{n}E_{c\times g}^{n} \end{matrix} \right)\approx F_{n\times c}S_{c\times g}.$$

Therefore, if we want to estimate the cell type proportion matrix based on those signature genes, we have

$$F={BS^{T}(SS^{T})}^{-1}=\left( X\beta+T \right)S^{T}\left( SS^{T} \right)^{-1}$$

$=X\beta S^{T}\left( SS^{T} \right)^{-1}+TS^{T}\left( SS^{T} \right)^{-1}$.

Only the first part of the equation is affected by genetic variants in the equation above, then the genetic component of cell type proportions can be represented as $X\beta S^{T}\left( SS^{T} \right)^{-1}$. Besides, because we do not know the exact eQTL effect size $\beta$, we use the estimated cis-eQTL effects  $\hat{\beta}$ as the proxy.

Due to the limited sample size in most eQTL studies, only cis-eQTL effects are reported. However, both cis-SNPs and trans-eQTLs may affect cell type proportion $F$ in $F=X\beta S^{T}\left( SS^{T} \right)^{-1}+TS^{T}\left( SS^{T} \right)^{-1}$. We would like to investigate whether cis-eQTL actually contribute to genetic component of cell type proportions. Using bulk-level expression matrix and signature matrix, we estimated the cell type proportions for GTEx samples using CIBERSORT. Using the estimated cell type proportions as phenotypes, we got the GWAS summary statistics of different cell type proportions. Using cis-eQTLs of signature genes in the corresponding tissues as annotations, we investigated whether the heritability of cell type proportions is enriched in cis-eQTLs of signature genes. In 179 (49.8%) of 359 cell type proportions across 30 tissues, their cell type proportion heritability was enriched for cis-eQTL of signature genes in the corresponding cell type (heritability fold-change larger than 1 in cis-eQTL regions compared to other regions). Due to limited sample size, only 13 (7.2%) of these 179 cell type proportions had significant heritability enrichment (fold change p value < 0.05 before multiple test correction) for the corresponding cis-eQTLs. These results suggest that the cell type proportion heritability is enriched for cis-eQTLs for signature genes, therefore the contribution of cis-eQTLs to cell type proportions. Admittedly due to the limited sample size in cell type proportion analysis, the enrichment results were not statistically significant especially after multiple test correction. We will perform cell type proportion heritability enrichment analysis when more data become available.

Similarly due to limited power in inferring and estimating trans-eQTL effects, we only consider cis-eQTL effects in the cWAS model. The cWAS test statistics is

$$z_{c}\approx\sum_{p} sd\left( X_{p} \right)z_{p}M_{c,p}/sd(\hat{F_{c}})$$

$$M_{c,p}=\hat{\beta}_{t,p}S_{t}A_{c}$$

where $z_{c}$ is the cWAS test statistics for cell type c; $\hat{\beta}_{t,p}$ are the eQTL effects of SNP $p$ for signature genes in tissue $t$, where we only consider cis-eQTL effects here. When we consider both trans- and cis-eQTL effects here, the test statistics will become

$$z_{c}\approx\sum_{p} sd\left( X_{p} \right)z_{p}\left( \hat{\beta}_{t,p,cis}+\hat{\beta}_{t,p,trans} \right)S_{t}A_{c}/sd(\hat{F_{c}})$$

$$=\sum_{p} \frac{sd\left( X_{p} \right)z_{p}{\hat{\beta}_{t,p,cis}S}_{t}A_{c}}{sd\left( \hat{F_{c}} \right)}+\sum_{p} \frac{sd\left( X_{p} \right)z_{p}{\hat{\beta}_{t,p,trans}S}_{t}A_{c}}{sd\left( \hat{F_{c}} \right)}$$

We can see that in the equation above, our current cWAS test statistics that only considers cis-eQTL effects) will lead to lower statistical power if both cis- and trans- eQTL effects of the same SNP are of the same direction. We note that the trans-eQTL effects are likely smaller than cis-eQTL effects [1]. As a result, the second term of the above equation is smaller than the first term, which is the cWAS test statistics.

**Methods: cell type proportion QTLs**

For each tissue, we extracted the bulk expression levels from GTEx and estimated cell type compositions using CIBERSORT and the corresponding signature matrix curated from the HCL database (which was also used in other sections of the manuscript here). Further, we regressed the estimated cell type compositions/proportions over covariates like sex and PEER factors, which were also considered in eQTL analysis in the GTEx (v8) study.

Then for each tissue, we only considered cis-SNPs (1Mb downstream/upstream of gene transcription starting sites) of signature genes to identify cell type proportion QTLs. Then for each cell type, we calculated the genotype effects of each SNP on cell type proportions (after regressing out covariate effects like discussed above).

**References**

1. Võsa U, Claringbould A, Westra HJ, Bonder MJ, Deelen P, Zeng B, et al. Large-scale cis-and trans-eQTL analyses identify thousands of genetic loci and polygenic scores that regulate blood gene expression. Nat Genet. 2021 Sep;53(9):1300-10.
