# Supplementary material for: A statistical framework to identify cell types whose genetically regulated proportions are associated with complex diseases
Source: PLoS Genet. 2023 Jul 31;19(7):e1010825. doi: 10.1371/journal.pgen.1010825 (PMC10414598; doi:10.1371/journal.pgen.1010825)
Supplement: Attachment — Submitted filename: Reviewer_responses.docx [file pgen.1010825.s019.docx]

Reviewer #1: Liu et al. presented a novel analysis frame cWAS, cell type wide association study that integrates genetic data with transcriptomics data to identify cell types whose genetically regulated proportions (GRPs) are trait-associated. The manuscript is well written and the results are clearly presented. Here are some concerns of mine:

1. How much does the signature matrix affect gene expression? With different signature matrices of the same tissue type, do gene-regulated proportions have the same effect size to phenotypes or the same power?

Response: We thank the reviewer for the question. As the reviewer implied, different signature matrices will lead to different statistical power in identifying disease-associated genetically-regulated cell type proportions. More specifically, cWAS has lower statistical power with a smaller number of signature genes used in the analysis. With fewer signature genes considered in cWAS analysis, fewer SNPs would be considered in the burden test statistics in the last step of cWAS, which would lead to lower statistical power. This is consistent with our simulation results. now included in the main text (lines 169-172):

*“When only half of the signature genes were used, there was a significant drop in statistical power although the type I error was still well-controlled (Figure 2d). With an increasing proportion of signature genes used, there was improved power in identifying associated cell types (Figure 2a).”*

2. The genetically regulated cell type proportions F ̂_c does not reflect the true cell type proportions since (1) it is not normalized with sum-to-one; (2) the imputed bulk expressions are subject to the part that genetic variation can explain. Therefore, the genetically regulated cell type proportions reflect the projection of imputed expression to cell type-specific expression, which genetic variations can explain. Can you discuss more on genetically regulated cell type proportions from imputed gene expression compared to cell type proportions from deconvolution methods?

Response: We agree with the reviewer that the genetically regulated cell type proportion F_c does not reflect the true cell type proportions. We have added the following discussion in the main text (lines 405-410) of the main text:

“*Third, unlike many other cell type deconvolution methods, we did not impose the non-negativity constraint nor requiring the cell type proportions sum to 1. Utilizing GWAS summary statistics, it is technically intractable to get the closed form of a test statistic to quantify the effects of imputed cell type proportion under these constraints. The imputed expression levels were adjusted for covariates like age and sex. Therefore, the non-negative constraint is impractical since the imputed expression is not always positive.”*

Also, we have also investigated the relationship between genetically regulated cell type proportions and cell type proportions from deconvolution methods. First, utilizing the oneK1k dataset ([1], N=982, which has matched individual-level genotype data and single cell expression data), we found that in peripheral blood, 4 out of 20 cell types have imputed genetically regulated cell type proportion that explained significant amount of variance of assayed cell type proportions (p value < 0.1). That is probably due to limited sample sizes and limited cis-expression heritability (mean h^2 of 0.1 in previous studies [2]). We have added the analysis results in the main text lines 413-417:

“*Utilizing the oneK1k dataset (N=982) in peripheral blood, we found that for 4 out of 20 cell types, the imputed genetically regulated cell type proportions explained significant amount of cell type proportion variances (p value <0.1). Even though the significance level is not that high, it can be partially due to limited sample sizes and limited cis-expression heritability (mean* $h^{2}$ *of 0.1 in previous studies).*”

Besides, comparing the cell type proportion from linear regression (w/o sum-to-1 constraints) with CIBERSORT-decomposed cell type proportions, we found that these two sets of proportions showed significant correlation in 78.6% of cell types with a mean correlation level of 0.36. We have added the following discussion (lines 410-413):

*“Also, empirically we found that the CIBERSORT estimated cell type proportions (with both non-negative and sum-to-1 constraints) showed significant correlations with the estimated cell type proportions from simple linear regression (without either constraint) in 78.6% of cell types (mean correlation: 0.36).”*

[1] Yazar S, Alquicira-Hernandez J, and et. al., Single-cell eQTL mapping identifies cell type-specific genetic control of autoimmune disease. Science. 2022 Apr 8;376(6589):eabf3041. doi: 10.1126/science.abf3041. Epub 2022 Apr 8. PMID: 35389779.

[2] Ouwens KG, Jansen R, and et. al., A characterization of cis- and trans-heritability of RNA-Seq-based gene expression. Eur J Hum Genet. 2020 Feb;28(2):253-263. doi: 10.1038/s41431-019-0511-5

3. There are deconvolution methods such as MuSiC (Wang et al. 2019), SCDC (Dong et al. 2020), and Bisque (Jew et al. 2020) that utilize all genes in bulk expression without marker gene selection. Aside from the signature matrix and signature genes selected from CIBERSORT, the well-known deconvolution method, can you discuss more why only include the signature genes in the model? What are the pros and cons of including more genes in cWAS study?

Response: We thank the reviewer for these questions. We agree with the reviewer that the genes used in the cWAS analysis will affect final association test results. To further get the exact effects of used genes in identifying cWAS associations, we utilized different fold changes and p value thresholds in identifying signature genes when applying cWAS in identifying disease-associated genetically regulated cell type proportions. We found that the number of cWAS-identified associations is significantly correlated with the number of genes used in cWAS analysis (p=6.67e-3, cor=0.14, supplementary table 10), which means that more genes utilized may lead to higher statistical power but also higher type-I error. In the meantime, some existing methods consider all genes for cell type proportion decomposition. Integrating all genes will also lead to potential noises (since some genes have large cross-subject variances and it is unreasonable to consider them for cell type proportions), which are mitigated by considering cross-subject variance of genes (MuSiC) or multiple datasets (SCDC, InteRD). Further extension of cWAS to consider more signature genes further boost statistical power. We have added the discussion in lines 419-426 of the main text:

*“We found that the number of cWAS-identified associations is significantly correlated with the number of genes used in cWAS analysis (p=6.67e-3, cor=0.14, supplementary table 10), which means that more genes utilized may lead to higher statistical power but also higher type-I error. Here we only consider signature genes in cWAS analysis to identify disease-associated genetically regulated cell type proportions. Some existing methods consider all genes for cell type proportion decomposition. Certain genes have large cross-subject variances and it is unreasonable to consider them for cell type proportions, thus integrating all genes may lead to potential noises, which are mitigated by considering cross-subject variance of genes (MuSiC) or multiple datasets (SCDC, InteRD). Further extension of cWAS to consider more genes is worth exploration.”*

Reviewer #2: This article proposes a statistical framework, called cWAS, which interprets GWAS findings in a cell type proportion manner. cWAS has been demonstrated and evaluated in both simulation and real-data analyses with higher statistical power detecting more significant results than existing methods. The identified disease-associated cell type proportions can furthermore serve as biological markers that could be useful to identify individuals with higher genetic risk. The idea of cWAS is interesting, the method is described well, and the data application results are convincing. The followings are some comments that may help further improve the manuscript:

1. More information is needed to describe the simulation study. For example, what does “600” replicates mean? Monte Carlo replicates, or sample size in each Monte Carlo run? It would be good to see how this method performs under different sample sizes. Whether does this method require substantial sample size to achieve desired power? This evaluation will be helpful and can provide guidance in practice.

Response: We thank the reviewer for the suggestions and questions. 600 replicates mean Monte Carlo replicates. Yes, good performance of cWAS does require substantial sample size as we have demonstrated in the supplementary text and Supplementary Figure 10. We have found that compared to GWAS sample size, the sample size for eQTL effect results have more significant effects in cWAS statistical power. We have added the detailed explanation in the supplementary text:

*“Compared to GWAS sample size (p=0.16), the eQTL sample size (p=3.6e-7) has more significant effects on the statistical power. This indicates that the imputation weights/models for bulk-level gene expression are more likely to limit the statistical power of cWAS models. Both the original test and the permutation-like test showed similar patterns. The top selection rate was more likely to be affected by GWAS sample size (p=9.7e-4) than the eQTL sample size (p=0.47). More importantly, for both the original test and the permutation-like test, when the sample size of eQTL studies was smaller than half of the sufficient sample size (n=670 in our simulation), the statistical power was even smaller than the corresponding FIR, indicating the important role of eQTL sample sizes in cWAS analysis.”*

To make the results of sample size effect clearer to the readers, we have added the explanation in the main text as well in lines 172-177:

*“Besides, based on our simulation studies, the eQTL study sample size has a more significant effect on the cWAS statistical power than the GWAS sample size (Supplementary Text). Compared to GWAS sample size (p=0.16), the eQTL sample size (p=3.6e-7) has more significant effects on the statistical power. This indicates that the imputation weights/models for bulk-level gene expression are more likely to limit the statistical power of cWAS models (S Fig. 10).”*

2. The authors should correct some inconsistent statements throughout the article: for example, on page 7, line 150, it says “When we simulated phenotypes independent of cell type proportions in the whole blood tissue (figure 2c)”. This does not match the information in the description of figure 2 and simulation section on page 25. Are they from blood tissue, or lung tissue? It is confusing.

Response: We thank the reviewer for careful reading and questions. For type-I power analysis, we simulated phenotypes based on genetically regulated cell type proportions of the lung tissue. For statistical power analysis, the phenotypes were simulated from genetically regulated cell type proportions of whole blood tissue. We have clarified that in the figure 2 description and simulation section on Page 26.

For figure 2 description: “*c) No cell type proportion in whole blood is associated with simulated phenotypes in this setting, and the test results shown here are those for whole blood genetically regulated cell type proportions. The simulated phenotypes are simulated as associated with genetically regulated cell type proportions in lung tissue. All signature genes in whole blood are known when conducting the cWAS test. The red dashed line indicates the 5% type I error. The green line indicates the proportion of simulations where any cell type in whole blood was selected as associated with the simulated disease status, the orange line indicates the proportion of simulations where M1 macrophages were selected as associated with the simulated disease. d) the same setting as that for c) and only 50% of signature genes in whole blood are known.”*

For simulation section on Page 26: “*For power analysis, we simulated phenotypes based on the imputed cell type proportion of M1 macrophages from whole blood tissue under different cis-eQTL heritability values from…*” and “*For the type-I error analysis, the disease phenotypes were simulated based on imputed proportions of basal cells in lung tissue*.”

3. Some notations in formulas are confusing. On page 22, line 478-480, gamma_t is used; however, on page 23, line 494, gamma_c is used. If gamma_c is one element in gamma_t vector, please clearly state it or make it in a more rigorous subscript. Also, on page 5, line 107, the equation is a bit weird and not consistent with the model on page 22. This also raises another concern about applying this method: are effects from all tissues estimated jointly by the model on page 22? any multiple test adjustment applied? More description is needed.

Response: We thank the reviewer for careful reading, questions and comments. We have added detailed explanation for the gamma_c and gamma_t in the main text and changed the equation/model on page 5, line 108 and stated that the proposed association test quantifies the marginal association between genetically-regulated cell type proportion of interests and disease/trait phenotype in line 108-121:

$$Y=\sum_{t} \sum_{c} \hat{F}_{\left( .,c \right),t}\gamma_{c,t}+\eta$$

“*where* $\gamma_{c,t}$ *is the effect of GRP for cell type* $c$ *of tissue* $t$ *on the trait, one element of the effect vector* $\gamma_{t}$ *of GRP of tissue* $t$*; and* $\eta$ *includes both genetic effects not mediated by genetically regulated cell type proportions and random noises.* $\hat{F}_{\left( .,c \right),t}$ *is the estimated GRPs of a cell type* $c$ *in tissue* $t$*, which is the* $c$*th column of the* $\hat{F}_{t}$ *matrix. However, individual-level genotype data are not always available for GWAS, which makes the direct estimation of* $\gamma_{c, t}$ *from the above two-step procedure infeasible. With only summary statistics available and assuming the effects* $\gamma_{c, t}$ *of all tissue* $t$ *and cell type* $c$ *are independent of each other , we propose to use the following approach to assessing the marginal association between GRPs of a cell type* $c$ *in tissue* $t$ *and traits*

$$z_{c,t}\approx\sum_{p} sd\left( X_{p} \right)z_{p} \hat{\beta}_{t,p}S_{t}\left( S_{t}^{T}S_{t} \right)_{.,c}^{-1}/sd(\hat{F}_{\left( .,c \right),t}),$$

*where* $sd(X_{p})$ *is the genotype standard deviation of SNP* $p$*, calculated from a reference panel;* $z_{p}$ *is the GWAS z score for SNP* $p$*;* $\hat{\beta}_{t,p}$ *is the imputed tissue-level gene expression vector of SNP* $p$ *across* $G$ *signature genes in tissue t;”*

We have also stated that the test results were corrected by the number of cell types in each tissue respectively.in lines 128-129:

*“Corresponding tissue-level multiple-test correction was applied for test statistics in each tissue respectively.”*

4. On page 22, the estimation of cell type proportion is based on the logic of simple linear model. Any specific reason for considering this simple estimation? How does this estimation ensure the property of sum up equal to one? There are many advanced deconvolution tools developed in recent years. More literature review in this area is highly suggested. Some relevant references are listed below:

“Bulk tissue cell type deconvolution with multi-subject single-cell expression reference (2019)”

“Omnibus and robust deconvolution scheme for bulk RNA sequencing data integrating multiple single-cell reference sets and prior biological knowledge (2022)”

Response: We thank the reviewer for these questions. Due to the limited availability of individual-level datasets with both gene expression, genotypes, and trait phenotypes, the cWAS test statistics are mainly for summary-stats level data. Therefore, it is non-trivial to apply the sum-to-1 or more sophisticated constraints in estimating genetically-regulated cell type proportions, but we 1) found that the correlation between linear-regression decomposed cell type proportion is significantly correlated with those decomposed from sophisticated methods like CIBERSOT (in 78.6% cell types, lines); 2) suggest that further work integrating constraints of cWAS method may help to unravel more biological insights. We have included the discussion the main text lines 405-418:

“Third, unlike many other cell type deconvolution methods, we did not impose the non-negativity constraint nor requiring the cell type proportions sum to 1. Utilizing GWAS summary statistics, it is technically intractable to get the closed form of a test statistic to quantify the effects of imputed cell type proportion under these constraints. The imputed expression levels were adjusted for covariates like age and sex. Therefore, the non-negative constraint is impractical since the imputed expression is not always positive. Also, empirically we found that the CIBERSORT estimated cell type proportions (with both non-negative and sum-to-1 constraints) showed significant correlation with estimated cell type proportions from simple linear regression (without either constraint) in 78.6% of cell types (mean correlation: 0.36). However, an alternative way to tackle those difficulties in the future will help to gain better biological insights.”

We agree with the reviewer that further literature review in the field is needed. We have added detailed explanation in the discussion part of the main text, lines 419-426:

*“We found that the number of cWAS-identified associations is significantly correlated with the number of genes used in cWAS analysis (p=6.67e-3, cor=0.14, supplementary table 10), which means that more genes utilized may lead to higher statistical power but also higher type-I error. Here we only consider signature genes in cWAS analysis to serve as a conservative approach to identify disease-associated genetically regulated cell type proportions. Previous methods consider all genes for cell type proportion decomposition. Certain genes have large cross-subject variances and it is unreasonable to consider them for cell type proportions, thus integrating all genes will also lead to potential random effects, which are mitigated by considering cross-subject variance of genes (MuSiC) or multiple datasets (SCDC, InteRD). Further extension of cWAS in considering more genes worth exploration.”*
